# HMOX1 drives dihydroartemisinin-sensitized ferroptosis antagonized by mitochondrial fusion

**DOI:** 10.1016/j.isci.2025.114382

**Published:** 2025-12-08

**Authors:** Zi-Jie Deng, Jing Zhang, Zhang-Zhong Yang, Qing-Zhang Tuo, Peng Lei

**Affiliations:** 1 Department of Neurology and State Key Laboratory of Biotherapy, National Clinical Research Center for Geriatrics, West China Hospital, Sichuan University, Chengdu 610041, P.R. China

**Keywords:** Drug delivery system, Drug dispensing, Pharmacology, Therapeutics

## Abstract

Artemisinin is the key component of artemisinin-based combination therapy (ACT) for malaria. Combinations of artemisinin with partner drugs demonstrate significant therapeutic potential in various diseases, including cancer. However, the precise mechanisms by which artemisinin, in combination with partner drugs, induces cell death are still not fully understood. Ferroptosis, a distinct form of cell death characterized by its dependence on iron, oxygen, and phospholipids (PLs), represents one potential pathway. In this study, we discovered that dihydroartemisinin (DHA), the active metabolite of artemisinin and its derivatives, sensitizes cells to ferroptosis induced by GPX4 inhibition. Through integrated data analysis and experimental validation, we found that DHA enhances ferroptosis sensitivity by promoting heme oxygenase 1 (HMOX1, HO-1)-mediated mitochondrial oxidative stress, thereby triggering a feedback loop that promotes mitochondrial fusion. These results broaden our understanding of the mechanisms of DHA in combination with partner drugs, and provide insights for clinical translation of ferroptosis.

## Highlights

• Non-lethal doses of DHA sensitize cells to GPX4-dependent ferroptosis
• DHA activates HMOX-1, forming a feedback loop that drives mitochondrial fusion via Mfn1/2
• Mitochondrial fusion acts as a suppressor of ferroptosis
• Low Mfn2 levels may correlate with longer overall survival in sarcoma and thymoma patients

## Graphical abstract

**Figure:**
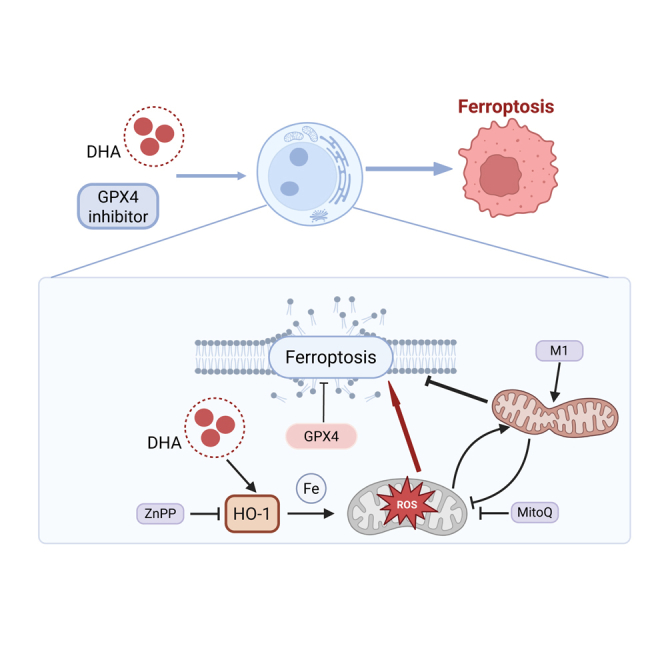

## Introduction

Artemisinin, derived from Chinese herbs, is a first-line treatment for malaria.^1^ Among the affected organs, cerebral malaria is a fatal complication and may result in long-term neurological sequelae, whose disease heterogeneity and mechanisms may differ due to the isolation provided by the blood-brain barrier.^2^ To enhance therapeutic efficiency and prevent drug resistance, artemisinin-based combination therapy (ACT), which combines short-acting artemisinin or its derivatives with a long-acting antimalarial partner drug, such as piperaquine and amodiaquine, is commonly applied in practice.^3^ Additionally, the combination of artemisinin with other drugs has been found effective for other diseases, particularly cancer.^4^ The pharmacological mechanism of artemisinin or ACT involves cell death induction^5^^,^^6^; however, the specific pathways remain elusive. It is reported that the cell death induced by artemisinin and its derivatives is highly dose-dependent, where a high dose directly induces cell toxicity.^7^^,^^8^ Therefore, treating artemisinin-resistant malaria by increasing the dose or prolonging the duration presents significant safety concerns.^9^ A lower dose of artemisinin or its derivatives may be combined with other drugs to provide a therapeutic window.

The key active chemical structure in artemisinin and its derivatives is the endoperoxide bridge, where its activation generates free radicals.^10^ In cells, mitochondria are the primary source of cellular oxidation,^11^ and mitochondrial dysfunction participates in cell death pathways, particularly in ferroptosis. Mitochondrial morphological damage is a common subcellular pathological feature in ferroptosis, and the glutathione (GSH)- Glutathione Peroxidase 4 (GPX4) pathway, the CoQ-ferroptosis-suppressor-protein 1 (FSP1) pathway, the tetrahydrobiopterin (BH4)-GTP cyclohydrolase 1 (GCH1) pathway, and the Dihydroorotate dehydrogenase (DHODH) pathway in mitochondria all participate in ferroptosis.^12^^,^^13^^,^^14^^,^^15^ As a distinct cell death from apoptosis, ferroptosis is modulated by iron, oxygen, and phospholipids (PLs)^16^^,^^17^^,^^18^ and has been implicated in various diseases.^19^^,^^20^^,^^21^^,^^22^^,^^23^ However, the precise function of mitochondrial redox homeostasis and quality control in ferroptosis remains poorly understood, and whether the activation of mitochondrial oxidation modulates ferroptosis remains highly controversial.^24^^,^^25^^,^^26^

In the present study, we aimed to investigate the effects of dihydroartemisinin (DHA), the active metabolite of artemisinin and its derivatives,^27^ in combination with cell death agonists and its pharmacological mechanism. We compared the effects of DHA in combination with different cell death agonists and examined the state of oxidative stress and mitochondrial function. Through screening and validation, we identified key regulatory proteins for the combinational effects and discovered new targets of ferroptosis. These data may provide further insights into the combination therapy of artemisinin and ferroptotic mechanisms.

## Results

### DHA specifically promotes GPX4-dependent ferroptosis

**Figure 1:**
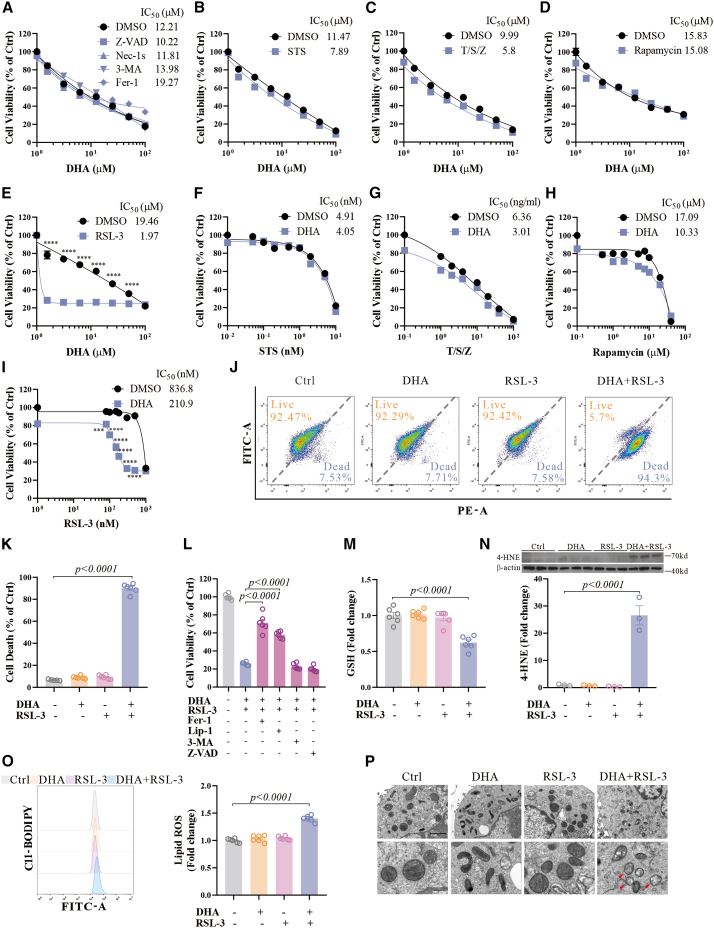
DHA specifically promotes GPX4-dependent ferroptosis (A) Cell viability of N27 cells treated with different doses of DHA for 48 h in the absence or presence of different cell death inhibitors (20 μM Z-VAD; 10 μM Nec-1s; 1 mM 3-MA; 1 μM Fer-1). (B–E) Cell viability of N27 cells treated with different doses of DHA for 48 h in the absence or presence of cell death inducers (1 nM STS; 1 ng/mL TNF-α (T), 10 nM SM-164 (S), and 20 μM Z-VAD; 5 μM rapamycin; 100 nM RSL-3). Two-way ANOVA was performed. (F–I) Cell viability of N27 cells treated with different doses of cell death inducers for 48 h in the absence or presence of 1.5 μM DHA (doses of T/S/Z cocktails were processed proportionally and displayed according to the dose of TNF-α). Two-way ANOVA was performed. (J and K) Live and dead staining assay of N27 cells treated with DHA (1.5 μM) and RSL-3 (100 nM) for 48 h. (L) Cell viability of N27 cells treated with DHA (1.5 μM) and RSL-3 (100 nM) for 48 h in the absence or presence of cell death inhibitors (1 μM Fer-1, 1 μM Lip-1, 0.5 mM 3-MA, 20 μM Z-VAD). (M) GSH levels in N27 cells treated with DHA (1.5 μM) and RSL-3 (100 nM) for 12 h were detected. (N) Western blot and quantifications of the 4-HNE and β-actin expression in N27 cells treated with DHA (1.5 μM) and RSL-3 (100 nM) for 12 h, *n* = 3 wells from one representative of two independent experiments. (O) N27 cells were treated with DHA (1.5 μM) and RSL-3 (100 nM) for 12 h, and lipid ROS was detected by C11-BODIPY using flow cytometry. Representative histograms for fluorescence of oxidized C11-BODIPY and the ratio of the MFI of oxidized to reduced C11-BODIPY are shown. (P) Transmission electron microscopy (TEM) of N27 cells treated with DHA (1.5 μM) and RSL-3 (100 nM) for 12 h. Red arrows indicate shrunken mitochondria. Scale bars: upper panel = 2 μm; lower panel = 500 nm, as indicated. Data are means ± SEM, *n* = 6 wells from one representative of two independent experiments unless specified. One-way ANOVA was performed unless specified.

DHA was reported to induce cell death with molecular features of apoptosis and autophagy in cancer cell lines.^28^^,^^29^ Considering that cerebral malaria is one of the most severe forms of malaria with pronounced neuronal damage, we first tested the nature of cell death induced by DHA in rat dopaminergic neuron N27 cells. The cell death induced by DHA (concentration range from 1.5 to 100 μM) could not be rescued by inhibitors of cell death including apoptosis inhibitor Z-VAD-FMK (Z-VAD), necroptosis inhibitor Necrostatin-1s (Nec-1s), autophagy inhibitor 3-methyladenine (3-MA), or ferroptosis inhibitor ferrostatin-1 (Fer-1) (Figure 1A). This is consistent with previous reports^30^^,^^31^ and the cell death observed following hydrogen peroxide exposure (Figure S1A), indicate that DHA causes non-programmed cell death associated with oxidative stress. However, when DHA was co-treated with different cell death agonists, namely apoptosis inducer staurosporine (STS), necroptosis inducer TNF-α (T), SM-164 (S), and Z-VAD-FMK (Z), autophagy inducer rapamycin, ferroptosis inducer RSL-3, ferroptosis was significantly and specifically sensitized by DHA (Figures 1B–1E). More importantly, DHA significantly promoted ferroptosis at a non-lethal dose (Figures 1F–1I), distinguishing the effect from DHA toxicity.

Indeed, co-treating non-toxic doses of DHA and RSL-3 synergistically resulted in significant cell death (Figures 1J and 1K). These observations can be replicated using a second GPX4 inhibitor, ML162 (Figures S1B and S1C), a GSH synthesis inhibitor Buthionine sulfoximine (BSO) (Figure S1D), or in other neuronal cells, such as mouse primary neurons or the STHdhQ7/Q7 cells (Figures S1E–S1G). Additionally, we observed increased sensitivity to RSL-3 with DHA co-treatment in other types of cancer cell lines, including ferroptosis non-sensitive MDA-MB-231 and U251 cells, and ferroptosis sensitive HT-1080 cells (Figures S1H–S1J). However, DHA was not able to promote ferroptosis induced by system Xc^−^ inhibitor erastin or lipid oxidation inducer Ferroptosis Inducer 2 (FINO2) (Figures S1K–S1L), suggesting that DHA may sensitize GPX4-dependent ferroptosis.

To confirm that co-treatment induced ferroptosis, we examined the effects of cell death inhibitors and found that only ferroptosis inhibitors, Fer-1 or liproxstatin-1 (Lip-1), but not other cell death inhibitors 3-MA or Z-VAD-FMK, could rescue co-treatment-induced cell death (Figure 1L). Consistently, the levels of GSH were decreased following the co-treatment (Figure 1M), with markedly elevated 4-Hydroxynonenal (4-HNE) levels (Figure 1N) and elevated lipid reactive oxygen species (ROS) levels (Figure 1O). The size of mitochondria was decreased, and the mitochondrial cristae disappeared (Figure 1P). These biochemical and pathological changes are in accordance with cells undergoing ferroptosis.

### DHA increases cell sensitivity to ferroptosis by modulating cellular oxidative stress

**Figure 2:**
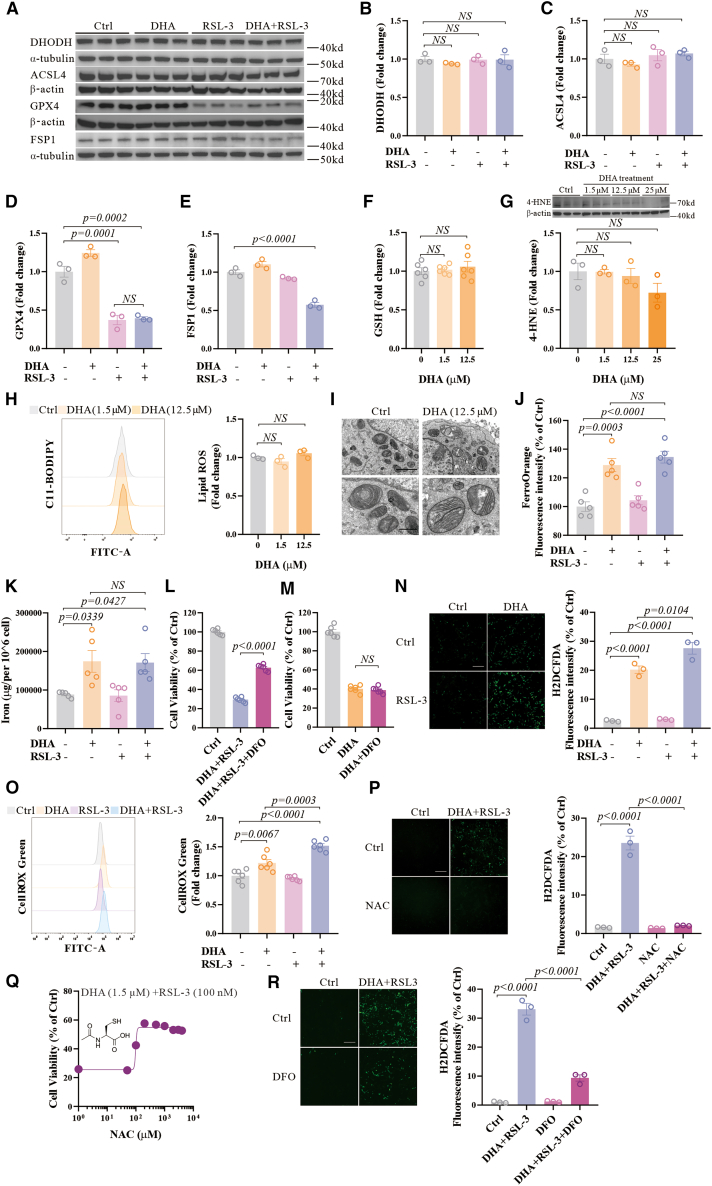
DHA increases sensitivity to ferroptosis by modulating cellular oxidative stress (A–E) Western blot and quantifications of the GPX4, ACSL4, FSP1, DHODH, β-actin, and α-tubulin expression in N27 cells treated with DHA (1.5 μM) and RSL-3 (100 nM) for 12 h. (F) GSH levels in N27 cells treated with different doses of DHA (0, 1.5 μM, 12.5 μM) for 12 h were detected, *n* = 6 wells from one representative of two independent experiments. (G) Western blot and quantifications of the 4-HNE and β-actin expression in N27 cells treated with different doses of DHA (0, 1.5, 12.5, and 25 μM) for 12 h. (H) N27 cells were treated with different doses of DHA (0, 1.5, and 12.5 μM) for 12 h and lipid ROS was detected by C11-BODIPY using flow cytometry. Representative histograms for fluorescence of oxidized C11-BODIPY and the ratio of the MFI of oxidized to reduced C11-BODIPY are shown. (I) TEM of N27 cells treated with DHA (12.5 μM) for 12 h. Red arrows indicate shrunken mitochondria. Scale bars: upper panel = 2 μm; lower panel = 500 nm, as indicated. (J) The average fluorescence intensity of FerroOrange in N27 cells treated with DHA (1.5 μM) and RSL-3 (100 nM) for 12 h, *n* = 5 wells from one representative of two independent experiments. (K) The iron levels detected by ICP-MS in N27 cells treated with DHA (1.5 μM) and RSL-3 (100 nM) for 12 h, *n* = 5 wells from one representative of two independent experiments. (L) Cell viability of N27 cells treated with DHA (1.5 μM) and RSL-3 (100 nM) in the absence or presence of DFO (50 μM) for 48 h, *n* = 6 wells from one representative of two independent experiments. (M) Cell viability of N27 cells treated with DHA (12.5 μM) in the absence or presence of DFO (50 μM) for 48 h, *n* = 6 wells from one representative of two independent experiments. (N) The representative images of H_2_DCFDA staining in N27 cells treated with DHA (1.5 μM) and RSL-3 (100 nM) for 12 h, and the average fluorescence intensity are shown. Scale bars, 200 μm, as indicated. (O) N27 cells were treated with DHA (1.5 μM) and RSL-3 (100 nM) for 12 h and detected by CellROX Green using flow cytometry. Representative histograms for fluorescence of CellROX Green and the average fluorescence are shown, *n* = 6 wells from one representative of two independent experiments. (P) The representative images of H_2_DCFDA staining in N27 cells treated with DHA (1.5 μM), RSL-3 (100 nM), and NAC (1 mM) for 12 h, and the average fluorescence intensity are shown. Scale bars, 200 μm, as indicated. (Q) Cell viability of N27 cells treated with NAC and DHA (1.5 μM) and RSL-3 (100 nM) for 48 h, *n* = 6 wells from one representative of two independent experiments. (R) The representative images of H_2_DCFDA staining in N27 cells treated with DHA (1.5 μM), RSL-3 (100 nM), and DFO (50 μM) for 12 h, and the average fluorescence intensity are shown. Scale bars, 200 μm, as indicated. Data are means ± SEM, *n* = 3 wells from one representative of two independent experiments unless specified. One-way ANOVA was performed.

We subsequently investigated the mechanisms by which DHA enhances the susceptibility of cells to ferroptosis. It is known that the interplay between redox signaling, lipid metabolism, and iron homeostasis regulates ferroptosis.^32^ We first examined the key proteins associated with lipid metabolism in ferroptosis, and found that the levels of acyl-CoA synthetase long chain family member 4 (ACSL4) and DHODH proteins remained unaltered (Figures 2A–2C). DHA-induced cell death was not accompanied by changes in GSH levels (Figure 2F), 4-HNE levels (Figure 2G), lipid ROS levels (Figure 2H), or altered morphology in mitochondria (Figure 2I), consistent with its insensitivity to Fer-1 (Figure 1A). These observations can be replicated in a second ferroptosis-sensitive neuronal cell line, the STHdhQ7/Q7 cell line (Figures S2A and S2B). Therefore, lipid metabolism may not account for the DHA-sensitized ferroptosis.

It was previously reported that DHA may promote ferroptosis by enhancing lysosome-mediated ferritin degradation and Iron Regulatory Protein (IRP)-mediated translational suppression of ferritin.^33^ We have found that intracellular iron levels were significantly elevated upon DHA treatment alone, and were not further affected by the co-treatment of DHA and RSL-3 (Figures 2J and 2K). However, chelation of iron effectively rescued cell death caused by co-treatment (Figure 2L), but cannot rescue DHA-induced cell death (Figure 2M). These results suggest that DHA does not directly induce ferroptosis but may promote it by increasing environmental iron levels.

Notably, we found that the level of oxidative stress was increased after DHA treatment and further increased with the co-treatment tested by microscopy and flow cytometry (Figures 2N and 2O), indicating that DHA may affect redox signaling systems. Both the elevated levels of cellular oxidative stress and cell death resulting from the co-treatment of DHA and RSL-3 were reversed by N-acetylcysteine (NAC) (Figures 2P and 2Q), a potent free radical scavenger.^34^ Both the level of GPX4 and FSP1 was decreased in the presence of DHA and RSL-3, consistent with ferroptosis (Figures 2A, 2D, and 2E).^12^ In addition, chelation of iron rescued the cellular ROS caused by co-treatment (Figure 2R). This implies that DHA-enhanced sensitivity to ferroptosis may be through intracellular oxidative stress mediated by iron.

### Activation of HO-1 by DHA promotes ferroptosis

**Figure 3:**
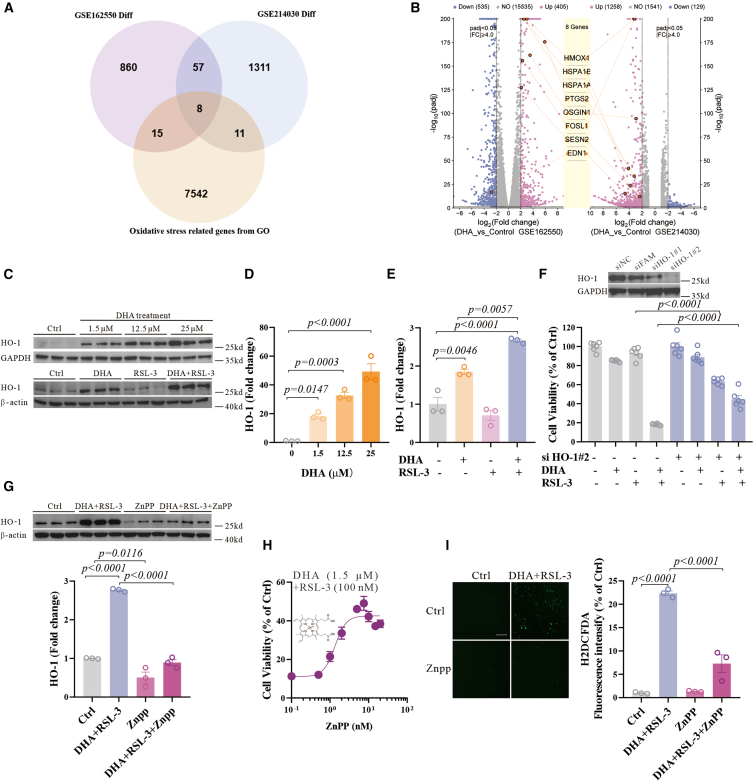
DHA promotes ferroptosis through the activation of HO-1 (A) Venn’s analysis used the data from the GSE162550 dataset and the GSE214030 dataset, and a set of genes related to oxidative stress from the Gene Ontology knowledgebase. Genes with an absolute log_2_ fold change greater than 2 (log_2_FC > 2 or log_2_FC < −2) and a *p* value <0.05 were considered significantly differentially expressed. (B) Volcano plot of the DEGs in the GSE162550 dataset and the GSE214030 dataset. (C–E) Western blot and quantifications of the HO-1, glyceraldehyde-3-phosphate dehydrogenase (GAPDH), and β-actin expression in N27 cells treated with different doses of DHA (0, 1.5, 12.5, and 25 μM) or treated with DHA (1.5 μM) and RSL-3 (100 nM) for 12 h. (F) N27 cells were transfected with two selected HO-1 siRNAs or a negative control siRNA, or FAM siRNA for 48 h, and the protein levels of HO-1 and GAPDH were detected by western blot. Cell viability of N27 cells treated with DHA (1.5 μM) and RSL-3 (100 nM) in the absence or presence of *HO-1* siRNA for 48 h, *n* = 6 wells from one representative of two independent experiments. (G) Western blot and quantifications of the HO-1 and β-actin expression in N27 cells treated with DHA (1.5 μM), RSL-3 (100 nM), and ZnPP (5 μM) for 12 h. (H) Cell viability of N27 cells treated with ZnPP and DHA (1.5 μM) and RSL-3 (100 nM) for 48 h, *n* = 6 wells from one representative of two independent experiments. (I) The representative images of H_2_DCFDA staining in N27 cells treated with DHA (1.5 μM), RSL-3 (100 nM), and ZnPP (5 μM) for 12 h, and the average fluorescence intensity are shown. Scale bars, 200 μm, as indicated. Data are means ± SEM, *n* = 3 wells from one representative of two independent experiments unless specified. One-way ANOVA was performed.

To illustrate the mechanisms, we analyzed data from existing transcriptomic data of DHA-treated cells (GSE162550 and GSE214030).^35^^,^^36^ The results of Kyoto Encyclopedia of Genes and Genomes (KEGG) and Gene Ontology (GO) enrichment analyses of differentially expressed genes (DEGs) highlighted pathways such as chemical carcinogenesis—reactive oxygen species, consistent with the findings associated with DHA-mediated oxidative stress (Figures S3A and S3B). We found eight shared genes related to oxidative stress in both datasets, which were identified by comparing the DEGs impacted by DHA. Among these genes, *HMOX1* (HO-1) exhibited the highest significant variability (Figures 3A and 3B).

HO-1 is a protein that generates free iron through heme degradation, known to regulate ferroptosis.^37^^,^^38^ HO-1 protein levels have changed following co-treatment with ferric ammonium sulfate (FAS) and DHA, and co-treatment further enhanced HO-1 expression, indicating a pivotal role for HO-1 in DHA-related iron metabolism (Figures S3C–S3E). We further examined the alterations in HO-1 protein levels and those of the homologous family member HMOX-2 (HO-2) in response to different doses of DHA treatment and found a dose-dependent increase in HO-1 protein levels, accompanied by a decrease in HO-2 levels (Figures 3C, 3D, S3F, and S3G). Subsequently, the level of HO-1 was elevated following co-treatment of DHA and RSL-3, while HO-2 remained unaltered (Figures 3C, 3E, S3F, and S3H). These data validated the transcriptomic analysis that HO-1 may be involved in DHA-sensitized ferroptosis.

We then genetically knocked down *HO -1* by small interfering RNA (siRNA), and observed a partial but significant rescue of ferroptosis induced by co-treatment of DHA and RSL-3 (Figure 3F). This implies that DHA facilitates RSL-3-induced ferroptosis, which may be regulated by HO-1. A competitive inhibitor of HO-1 enzymatic activity, zinc protoporphyrin (ZnPP),^39^ selectively reduced the level of HO-1 following the co-treatment of DHA and RSL-3 (Figure 3G), and subsequently inhibited the cell death (Figure 3H). ZnPP also mitigated the elevation in oxidative stress and iron levels following the co-treatment (Figures 3I and S3I). Hemin, an activator of HO-1, increased sensitivity to ferroptosis induced by RSL-3, mimicking the effect of DHA (Figures S3J and S3K). Co-treatment of hemin and RSL-3 resulted in a higher expression level of HO-1 compared to hemin alone (Figure S3L). The classical Keap1/Nrf2 signaling pathway, a well-established mechanism of HO-1 activation,^40^^,^^41^^,^^42^ was also investigated. However, neither proteins were altered after the co-treatment (Figures S3M–S3O). Therefore, HO-1 may be responsible for the cellular oxidative stress and cell death caused by DHA and RSL-3.

### DHA increases HO-1-dependent mitochondrial oxidative stress

**Figure 4:**
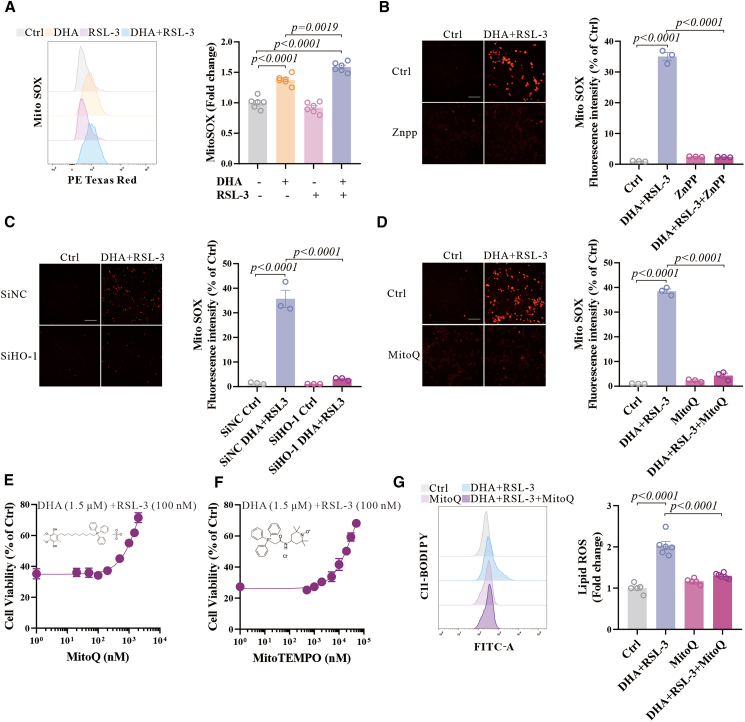
DHA facilitates ferroptosis by stimulating mitochondrial oxidative stress (A) N27 cells were treated with DHA (1.5 μM) and RSL-3 (100 nM) for 12 h and detected by MitoSOX using flow cytometry. Representative histograms and the average fluorescence are shown, *n* = 6 wells from one representative of two independent experiments. (B) The representative images of MitoSOX red staining in N27 cells treated with ZnPP (5 μM), DHA (1.5 μM), and RSL-3 (100 nM) for 12 h, and the average fluorescence intensity are shown. Scale bars, 200 μm, as indicated. (C) The representative images of MitoSOX red staining in N27 cells transfected with selected HO-1 siRNAs or negative control siRNA for 48 h, treated with DHA (1.5 μM), and RSL-3 (100 nM) for 12 h, and the average fluorescence intensity are shown. Scale bars, 200 μm, as indicated. (D) The representative images of MitoSOX red staining in N27 cells treated with MitoQ (5 μM), DHA (1.5 μM), and RSL-3 (100 nM) for 12 h, and the average fluorescence intensity are shown. Scale bars, 200 μm, as indicated. (E and F) Cell viability of N27 cells treated with MitoQ or Mito-TEMPO and DHA (1.5 μM) and RSL-3 (100 nM) for 48 h, *n* = 6 wells from one representative of two independent experiments. (G) N27 cells were treated with MitoQ (5 μM), DHA (1.5 μM), and RSL-3 (100 nM) for 12 h and lipid ROS was detected by C11-BODIPY using flow cytometry. Representative histograms for fluorescence of oxidized C11-BODIPY and the ratio of the MFI of oxidized to reduced C11-BODIPY are shown, *n* = 5 or 6 wells from one representative of two independent experiments. Data are means ± SEM, *n* = 3 wells from one representative of two independent experiments unless specified. One-way ANOVA was performed.

HO-1 is transported to mitochondria to induce oxidative stress,^11^^,^^43^ and therefore, we tested the level of mitochondrial ROS after the co-treatment with DHA and RSL-3. We found that it increased with DHA treatment alone and further increased after co-treatment (Figures 4A and S4A), consistent with changes in cellular oxidative stress. The HO-1 inhibitor ZnPP or the reduction of HO-1 levels by siRNA prevented the increase in mitochondrial oxidation caused by the co-treatment (Figures 4B and 4C). In addition, we found that mitochondria-targeted antioxidants MitoQ and Mito-TEMPO significantly mitigated mitochondrial oxidation (Figure 4D) and cell death (Figures 4E and 4F) triggered by DHA and RSL-3. The elevation in lipid ROS resulting from the co-treatment was also suppressed by MitoQ (Figure 4G). These findings indicated that mitochondrial oxidative stress induced by DHA is crucial for ferroptosis.

Furthermore, hemin also induced mitochondrial oxidation, with further exacerbation of the co-treatment of hemin and RSL-3, in accordance with the effect of DHA (Figure S4B). Mitochondrial-targeted antioxidants mitoquinone (MitoQ) and Mito-TEMPO rescued the cell death induced by co-treatment of hemin and RSL-3 (Figures S4C and S4D), indicating that HO-1 is crucial in DHA-promoted ferroptosis.

### Mitochondrial oxidative stress activates mitochondrial fusion

**Figure 5:**
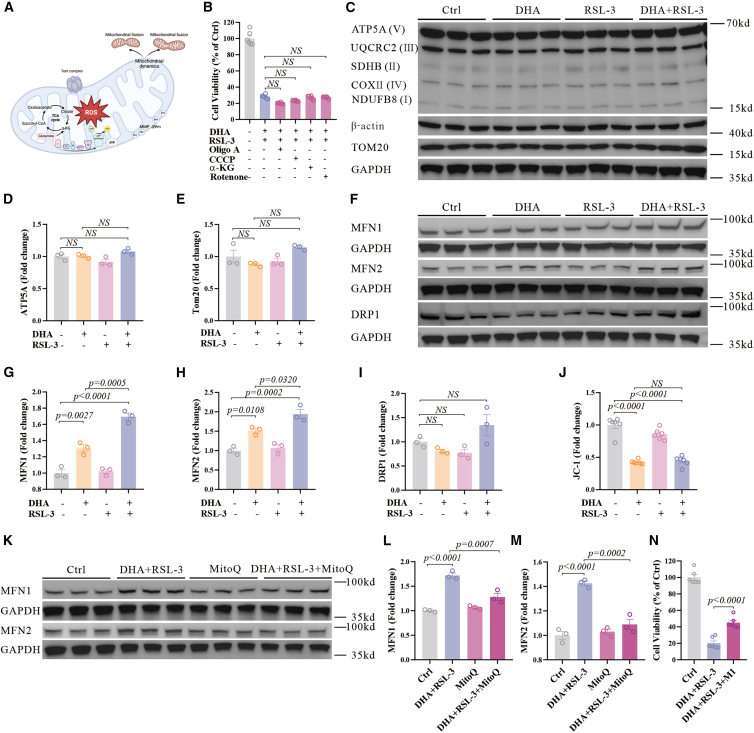
Mitochondrial oxidative stress induced by DHA and RSL-3 activates mitochondrial fusion (A) The thumbnail sketch of mitochondrial functions that may be regulated by mitochondrial oxidation. (B) Cell viability of N27 cells treated with DHA (1.5 μM) and RSL-3 (100 nM) for 48 h in the absence or presence of mitochondrial regulators (2 μM oligo A, 2 μM CCCP, 10 μM αKG, 1 μM rotenone), *n* = 6 wells from one representative of two independent experiments. (C–E) Western blot and quantifications of the OXPHOS, Tom20, β-actin, and GAPDH expression in N27 cells treated with DHA (1.5 μM) and RSL-3 (100 nM) for 12 h. (F–I) Western blot and quantifications of the MFN1, MFN2, DRP1, and GAPDH expression in N27 cells treated with DHA (1.5 μM) and RSL-3 (100 nM) for 12 h. (J) N27 cells were treated with DHA (1.5 μM) and RSL-3 (100 nM) for 12 h and detected by JC-1 using flow cytometry. Statistical analysis of the ratio of the MFI of JC-1 red to JC-1 green is shown, *n* = 6 wells from one representative of two independent experiments. (K–M) Western blot and quantifications of the MFN1, MFN2, and GAPDH expression in N27 cells treated with MitoQ (5 μM), DHA (1.5 μM), and RSL-3 (100 nM) for 12 h. (N) Cell viability of N27 cells treated with DHA (1.5 μM) and RSL-3 (100 nM) in the absence or presence of mitochondrial fusion promoter M1 (5 μM) for 48 h, *n* = 6 wells from one representative of two independent experiments. Data are means ± SEM, *n* = 3 wells from one representative of two independent experiments unless specified. One-way ANOVA was performed unless specified.

The redox homeostasis of mitochondria plays a pivotal role in the maintenance of normal mitochondrial function^44^^,^^45^ (Figure 5A). The mitochondrial oxidative phosphorylation process is a major source of mitochondrial oxidation, while previous studies have reported that it is associated with ferroptosis.^46^ We surveyed the effects of different pharmacological modulators of oxidative phosphorylation, including ATP synthase inhibitors oligomycin A, mitochondrial uncouplers CCCP, TCA cycle metabolite immediately downstream of glutaminolysis α-ketoglutaric acid (αKG), and mitochondrial complex I inhibitors rotenone on the co-treatment of DHA and RSL-3, and found that none of these inhibitors prevented cell death (Figure 5B). In addition, the impact of the co-treatment of DHA and RSL-3 on electron transport chain proteins, as well as the mitochondrial transporter receptor (Tom20), was limited, as no significant changes in protein levels were observed after co-treatment (Figures 5C–5E). This finding indicates that the treatment specifically affects mitochondrial dynamics without causing general degradation of key mitochondrial components or altering mitochondrial biogenesis.

Mitochondrial fusion and fission-mediated mitochondrial dynamics are crucial for the removal of damaged mitochondria and the maintenance of normal mitochondrial morphology.^44^ We then detected the effects of co-treatment of DHA and RSL-3 on mitochondrial fusion key regulators, mitochondrial fusion protein 1 (Mfn1) and mitochondrial fusion protein 2 (Mfn2), as well as mitochondrial fission central mediator dynamin-related protein 1 (Drp1). Both Mfn1 and Mfn2 were increased by DHA treatment, and further increased by co-treatment of DHA and RSL-3 (Figures 5F–5H), consistent with the level of mitochondrial oxidation. Both proteins exhibited a gradual increase over time (Figures S5A–S5C), suggesting a progressive activation of the mitochondrial fusion in response to sustained oxidative stress. In contrast, Drp1 was not changed significantly (Figures 5F and 5I). Consistently, we found a polarization of the mitochondrial membrane potential, essential for mitochondrial fusion,^47^ after both DHA treatment as well as co-treatment of DHA and RSL-3 (Figure 5J). Mitochondrial antioxidant, MitoQ, attenuated the upregulation of Mfn1 and Mfn2 levels (Figures 5K–5M). An inducer of mitochondrial fusion, the mitochondrial fusion promoter (M1), mitigated cell death caused by co-treatment (Figure 5N). These observations suggest that the co-treatment specifically modulates mitochondrial fusion.

### Mitochondrial fusion inhibits ferroptosis with clinical implications

**Figure 6:**
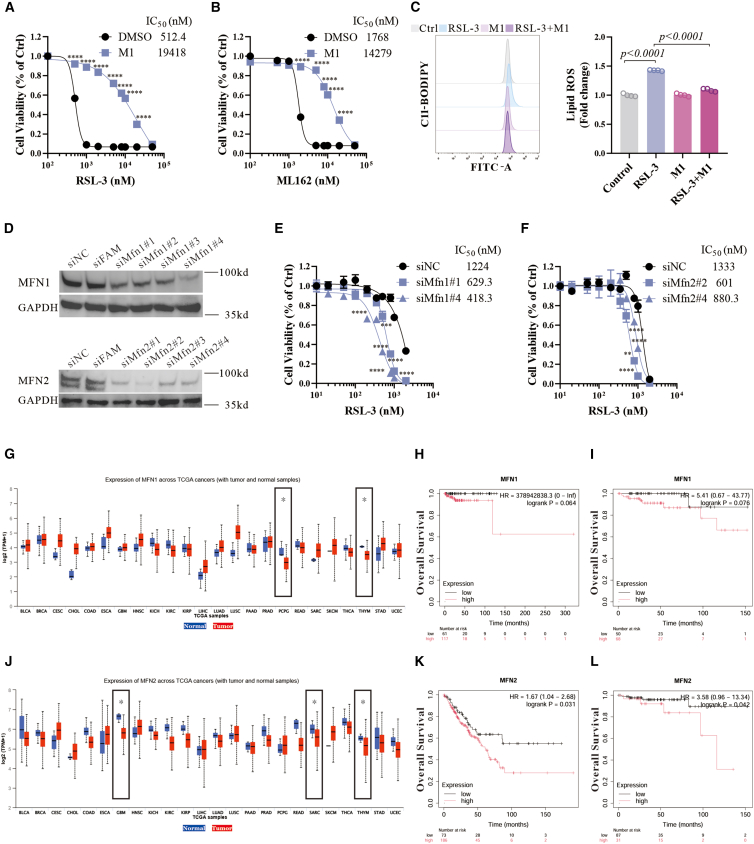
Mitochondrial fusion inhibits ferroptosis with clinical implications (A and B) Cell viability of N27 cells treated with RSL-3 or ML162 and M1 (2 μM) for 24 h, *n* = 6 wells from one representative of two independent experiments. Two-way ANOVA was performed. (C) N27 cells were treated with RSL-3 (1,000 nM) and M1 (2 μM) for 3 h and lipid ROS was detected by C11-BODIPY using flow cytometry. Representative histograms for fluorescence of oxidized C11-BODIPY and the ratio of the MFI of oxidized to reduced C11-BODIPY are shown, *n* = 4 wells from one representative of two independent experiments. (D) N27 cells were transfected with four selected *Mfn1* or *Mfn2* siRNAs and a negative control siRNA or FAM siRNA for 48 h, and the protein levels of Mfn1 and Mfn2 were detected by western blot. (E and F) Cell viability of N27 cells treated with RSL-3 in the absence or presence of *Mfn1* or *Mfn2* siRNA for 24 h, *n* = 6 wells from one representative of two independent experiments. Two-way ANOVA was performed. (G) Online UALCAN analysis of the expression levels of *Mfn1* across The Cancer Genome Atlas (TCGA) cancers. (H) Online Kaplan-Meier plotter analysis of pheochromocytoma and paraganglioma cancer patient outcomes. Differences in OS were compared in groups divided by *Mfn1* expression. (I) Online Kaplan-Meier plotter analysis of thymoma cancer patient outcomes. Differences in OS were compared in groups divided by *Mfn1* expression. (J) Online UALCAN analysis of the expression levels of *Mfn2* across TCGA cancers. (K) Online Kaplan-Meier plotter analysis of sarcoma cancer patient outcomes. Differences in OS were compared in groups divided by *Mfn2* expression. (L) Online Kaplan-Meier plotter analysis of thymoma cancer patient outcomes. Differences in OS were compared in groups divided by *Mfn2* expression. Data are means ± SEM; one-way ANOVA was performed unless specified.

It is known that the quality control and dynamic regulation of mitochondria regulate the redox imbalance of ferroptosis.^48^ We investigated whether mitochondrial fusion acts as a regulator of ferroptosis independent of DHA-RSL-3 co-treatment. M1 inhibited the cell death caused by ferroptosis inducer RSL-3 or ML162 (Figures 6A and 6B), and the lipid peroxidation triggered by RSL-3 (Figure 6C). In addition, reducing Mfn1 and Mfn2 levels by siRNA increased the sensitivity of ferroptosis (Figures 6D–6F). Therefore, Mfn1 and Mfn2-mediated mitochondrial fusion, which we initially observed with the co-treatment of DHA and RSL-3, inhibit ferroptosis.

These mechanistic insights may have clinical implications. Using an existing clinical dataset, UALCAN,^49^ we found reduced expression of *Mfn1* in tumors, e.g., pheochromocytoma and paraganglioma, and thymoma, compared to normal tissues (Figure 6G). Online Kaplan-Meier plotter analysis indicated a non-significant trend toward association between expression and overall survival (OS) in both tumor types (Figures 6H and 6I).^50^ Furthermore, UALCAN analysis showed that *Mfn2* was lower in glioblastoma multiforme, sarcoma, and thymoma (Figure 6J), and the higher level of *Mfn2* was correlated with poorer OS in patients with sarcoma and thymoma (Figures 6K and 6L). These results highlight the potential of targeting mitochondrial fusion or employing the co-treatment of DHA and RSL-3 in these tumors.

## Discussion

**Figure 7:**
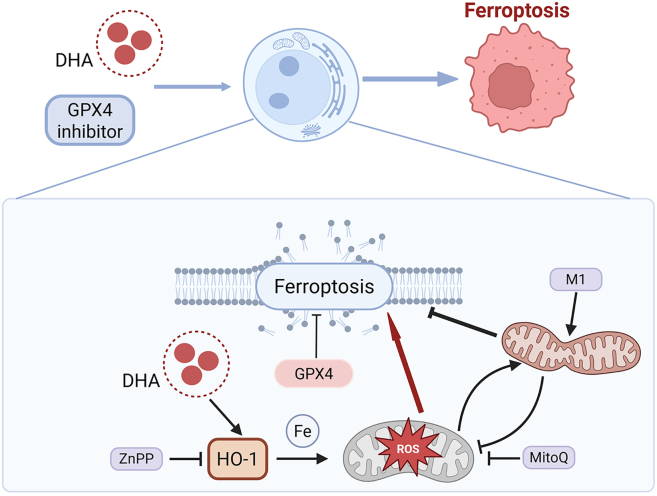
Schematic hypothesis

The combination of artemisinin with other drugs has been effective in a wide range of diseases, where the precise mechanisms of cell death induction remain unclear. Here, we report that non-lethal doses of DHA significantly promote ferroptosis by mitochondrial fusion suppression, a process regulated by mitochondrial oxidation through HO-1. Regulation of mitochondrial fusion alone inhibits ferroptosis, and it may be targeted for cancer therapy (Figure 7). This study provides new mechanistic insights into the therapeutic effects of DHA and identifies mitochondrial fusion as a new regulatory mechanism of ferroptosis, both of which shed light on future clinical studies.

Mitochondrial dynamics is a highly dynamic regulatory process, with mitochondrial fusion facilitating the mixing and exchange of mitochondrial contents, crucial for maintaining cellular homeostasis.^51^ However, while the involvement of mitochondria in ferroptosis is well supported by the evidence, the role of mitochondrial fusion in ferroptosis is contradictory. For instance, selenium was reported to promote mitochondrial fusion to rescue ferroptosis,^52^ but STRING activation-mediated mitochondrial fusion promoted ferroptosis.^53^ Here, we found that inhibition of mitochondrial fusion increased cell susceptibility to ferroptosis, consistent with two recent studies.^54^^,^^55^ In ferroptosis, mitochondria act as central hubs for energy production and oxidative stress generation, and fusion-mediated maintenance of mitochondrial integrity helps to prevent the onset of lipid peroxidation. The upregulation of Mfn1 and Mfn2 after co-treatment that we observed, therefore, may be a protective response. Our findings support that mitochondrial dynamics, particularly fusion, play a critical role in ferroptosis.

In recent years, DHA was reported to induce ferroptosis through various mechanisms.^56^^,^^57^^,^^58^ However, we did not observe that DHA directly induced typical characteristics of ferroptosis, but it resembled cell death by excess hydrogen peroxide. This discrepancy may be due to variations in experimental conditions, particularly the pronounced distinctions between neural and peripheral cells. Consequently, in the subsequent experiments, we employed a non-lethal dose of DHA that did not result in cell death to elucidate the mechanism by which it causes cell death in combination with ferroptosis inducers. On the other hand, we found here DHA promotes cell death induced by RSL-3, ML162, and BSO, but not by erastin or FINO2, suggesting that DHA promotes GPX4-dependent ferroptosis. The absence of an enhanced effect with co-treatment of DHA and FINO2 aligns with our observation that DHA alone does not directly trigger ferroptosis.

Previous studies have reported that artemisinin and its derivatives, including DHA, regulate HO-1 primarily through modulation of the Nrf2 pathway, with a focus on overall oxidative stress and inflammation.^59^^,^^60^^,^^61^^,^^62^^,^^63^^,^^64^^,^^65^ In contrast, we showed that DHA regulates HO-1 independently of the Keap1-Nrf2 pathway, regulated by the mitochondria-associated oxidative pathway driven by iron. These discrepancies may be attributed to the use of non-lethal low doses in our experimental setting, and the potential differences between peripheral and central regulatory mechanisms.^66^

We found here that the combination of non-lethal doses of DHA and RSL-3 induced significant cell death, where ferroptosis and mitochondria fusion inhibition play a role. It indicates its promising therapeutic potential for cancer, supported by the observations that *Mfn1* and *Mfn2* expression in several different types of cancer was reduced. The significant association between high *Mfn2* expression and poorer OS in patients with sarcoma and thymoma suggests that inhibition of mitochondrial fusion may represent a potential therapeutic strategy.

In conclusion, this study provides mechanistic insights for understanding and application of DHA in combination with other drugs to induce ferroptosis, which may be of clinical relevance.

### Limitations of the study

This study provides new insights into the cell death mechanisms of DHA in a set of cell culture experiments. Further validation in animal models of cerebral malaria may be necessary. Additionally, the research demonstrates the clinical potential of targeting mitochondrial fusion for treating specific tumor types. Considering the possibility of reverse causality or confounding factors, the clinical association requires further validation.

## Resource availability

### Lead contact

Further information and requests for resources and reagents should be directed to and will be fulfilled by the lead contact, Dr. Peng Lei (peng.lei@scu.edu.cn).

### Materials availability

All reagents and materials are listed in the key resources table. Materials are available on reasonable request.

### Data and code availability

• All data reported in this paper will be shared by the lead contact upon request.
• This paper does not report original code.
• Any additional information required to reanalyze the data reported in this paper is available from the lead contact upon request.

## Acknowledgments

This work was supported by the Scientific Research Innovation Capability Support Project for Young Faculty (ZYGXQNJSKYCXNLZCXM-H14), the Central Guidance for Local Science and Technology Development projects (no. 2023ZYDF074), the National Clinical Research Center for Geriatrics, West China Hospital, 10.13039/501100004912Sichuan University (Z2024JC007), Sichuan Science and Technology Program (2024YFHZ0010), and the 10.13039/501100013365West China Hospital 1.3.5 project for disciplines of excellence (ZYYC23016).

## Author contributions

Z.-J.D., investigation, data curation, and writing – original draft. J.Z. and Z.-Z.Y., investigation, data curation. Q.-Z.T., supervision, resources, and funding acquisition. P.L., conceptualization, funding acquisition, supervision, and writing – review & editing.

## Declaration of interests

The authors declare no competing interests.

## STAR★Methods

### Key resources table

**Table:** 

REAGENT or RESOURCE	SOURCE	IDENTIFIER
**Antibodies**		

Anti-4 Hydroxynonenal antibody (1:3000)	Abcam	ab46545; RRID:AB_722490
Anti-Glutathione Peroxidase 4 antibody (1:5000)	Abcam	ab125066; RRID:AB_10973901
Anti-FACL4 antibody (1:5000)	Abcam	ab155282; RRID:AB_2714020
AIFM2/FSP1 Polyclonal antibody (1:5000)	Proteintech	20886-1-AP; RRID:AB_2878756
DHODH Polyclonal antibody (1:5000)	Proteintech	14877-1-AP; RRID:AB_2091723
KEAP1 Monoclonal antibody (1:4000)	Proteintech	60027-1-Ig; RRID:AB_2132623
Anti-Nrf2 Antibody (1:2000)	HUABIO	R1312-8; RRID:AB_3073268
HO-1/HMOX1 Monoclonal antibody (1:5000)	Proteintech	66743-1-Ig; RRID:AB_2882091
HMOX2 Polyclonal antibody (1:1000)	Proteintech	14817-1-AP; RRID:AB_2118843
MFN1 Polyclonal antibody (1:1000)	Proteintech	13798-1-AP; RRID:AB_2266318
MFN2 Polyclonal antibody (1:5000)	Proteintech	12186-1-AP; RRID:AB_2266320
DRP1 (C-terminal) Polyclonal antibody (1:5000)	Proteintech	12957-1-AP; RRID:AB_2093525
Total OXPHOS Rodent WB Antibody Cocktail (1:1000)	Abcam	ab110413; RRID:AB_2629281
TOM20 Polyclonal antibody (1:5000)	Proteintech	11802-1-AP; RRID:AB_2207530
Anti-β-Actin (ACTB) Antibody (1:10000)	Sigma-Aldrich	A5441; RRID:AB_476744
HRP-conjugated GAPDH Monoclonal antibody (1:10000)	Proteintech	HRP-60004; RRID:AB_2737588
Alpha Tubulin Monoclonal antibody (1:10000)	Proteintech	66031-1-Ig; RRID:AB_11042766
Anti-Mouse IgG (whole molecule)–Peroxidase antibody produced in rabbit (1:10000)	Sigma-Aldrich	A9044; RRID:AB_258431
Anti-Rabbit IgG (whole molecule)–Peroxidase antibody produced in goat (1:10000)	Sigma Aldrich	A0545; RRID:AB_257896

**Chemicals, peptides, and recombinant proteins**		

Dihydroartemisinin	Cayman	19846
3-Methyladenine	Selleck	S2767
Z-VAD-FMK	Selleck	S7023
Necrostatin-1s	Selleck	S8641
Ferrostatin-1	Selleck	S7243
(1S,3R)-RSL3 (RSL3)	Selleck	S8155
Staurosporine	Selleck	S1421
Rapamycin	Selleck	S1039
Recombinant Human TNF alpha	Novoprotein Scientific	No. C008
SM-164	Selleck	S7089
Liproxstatin-1	Selleck	S7699
ML162	Absin	abs823477
N-acetylcysteine	Selleck	S1623
Deferoxamine mesylate	Selleck	S5742
Zinc Protoporphyrin	MedChemExpress	HY-101193
Hemin	Sigma-Aldrich	51280
Mitoquinone mesylate	Selleck	S8978
Mito-TEMPO	Selleck	S9733
Mitochondrial Fusion Promoter M1	Selleck	S3375
Oligomycin A	Selleck	S1478
CCCP	Selleck	S6494
α-Ketoglutaric acid sodium salt	Sigma-Aldrich	K1875
Rotenone	Selleck	S2348
H_2_O_2_	Macklin	H792072
FINO2	Selleck	E1244
BSO	Selleck	S9728
Erastin	Selleck	S7242
Ammonium iron(II) sulfate hexahydrate (FAS)	Sigma-Aldrich	215406
Cell Counting Kit-8 (CCK-8)	Selleck	B34304

**Critical commercial assays**		

Live and Dead Cell Assay	Abcam	ab115347
GSH and GSSG Assay Kit	Beyotime	S0053
BODIPY™ 581/591 C11	Thermo Fisher Scientific	D3861
H2DCFDA	MedChemExpress	HY-D0940
CellROX™ Green	Thermo Fisher Scientific	C10444
FerroOrange	Dojindo	F374
MitoSOX Red	Thermo Fisher Scientific	M36008
MitoProbe JC-1 Assay Kit	Thermo Fisher Scientific	M34152

**Experimental models: Cell lines**		

Rat: N27 cell line	From Dr. Ashley I. Bush	N/A
Mouse: STHdhQ7/Q7 cell line	From Dr. Boxun Lu	N/A
Human: HT-1080 cell line	Chinese Academy of Sciences cell library	TCHu170
Human: MDA-MB-231 cell line	Chinese Academy of Sciences cell library	SCSP-5043
Human: U251 cell line	Chinese Academy of Sciences cell library	SCSP-559

**Oligonucleotides**		

Rat HO-1 siRNA#1: 5′-GCCUCCUUGUACCAUAUCUTT-3′	This paper	N/A
Rat HO-1 siRNA#2: 5′-CCACACAGCACUACGUAAATT-3′	This paper	N/A
Rat Mfn1 siRNA#1: 5′- GCAGCAGUUUGUAAACUAUTT -3′	This paper	N/A
Rat Mfn1 siRNA#2: 5′-GUGGCAAACUCGGAAUCAATT-3′	This paper	N/A
Rat Mfn1 siRNA#3: 5′-GUGCACCAGUGAAGUCAAUTT-3′	This paper	N/A
Rat Mfn1 siRNA#4: 5′-GGAGAUACAGGGCUACAGATT-3′	This paper	N/A
Rat Mfn2 siRNA#1: 5′-GGUUUAUUGUCUUGAAAUGTT-3′	This paper	N/A
Rat Mfn2 siRNA#2: 5′-GGAAGAGCACCGUGAUCAATT-3′	This paper	N/A
Rat Mfn2 siRNA#3: 5′-ACGAGUACCAGAUGGACUUTT-3′	This paper	N/A
Rat Mfn2 siRNA#4: 5′-GCAGCAAGACAUGAUAGACTT-3′	This paper	N/A

**Software and algorithms**		

GraphPad Prism 8.0	GraphPad Software	https://www.graphpad.com/features
ImageJ ver 1.52a	ImageJ software	https://imagej.net/ij/features.html
FlowJo v10	FlowJo software	https://www.flowjo.com/

### Experimental model details

#### Primary neuronal culture

Primary neurons from the cortices were prepared as previously described.^67^ Cortices were harvested and dissociated using trypsin (Sigma, T-4665, USA). Dissociated neurons were seeded at a density of 6 × 10^5^ cells/mL in plating medium (DMEM with 10% fetal bovine serum, 5% horse serum, and 10 mg/L gentamycin sulfate). After incubation for 2 h, the medium was replaced with Neurobasal medium supplemented with B27, 500 μM GlutaMAX, and 10 μg/mL gentamycin sulfate (Thermo Fisher Scientific or Sigma, USA). Primary neurons were plated into 96-well plates for 7 days and treated with the selected compounds for 48 h before harvest.

#### Cell lines

The neuronal cell line N27 (a gift from Dr. Ashley I. Bush, The University of Melbourne), derived from E12 rat mesencephalic tissue (Merck, Bayswater), was cultured in RPMI 1640 (Gibco, Thermo Fisher Scientific) supplemented with 10% fetal bovine serum (NATOCOR, Argentina, SFBE) and 1% penicillin/streptomycin (Thermo Fisher Scientific) in a humidified incubator with 5% CO_2_. The neuronal cell line STHdhQ7/Q7 (a gift from Dr. Boxun Lu, Fudan University); the HT-1080 cell line (obtained from the Chinese Academy of Sciences cell library, Cat No. TCHu170); the MDA-MB-231 (obtained from the Chinese Academy of Sciences cell library, Cat No. SCSP-5043); the U251 cell line (obtained from the Chinese Academy of Sciences cell library, Cat No. SCSP-559) were grown in DMEM Medium with 10% fetal bovine serum (NATOCOR, Argentina, SFBE) and 1% penicillin/streptomycin (Thermo Fisher Scientific) in a humidified incubator with 5% CO_2_. These cell lines were authenticated before via Procell Life Science & Technology Co., Ltd. The cells are free from mycoplasma contamination.

### Method details

#### Reagents

The reagents used in the study are listed in the key resources table.

#### Cell viability assays

Cells were seeded into 96-well plates (5000 cells per well) and treated with the selected compounds after plating. Cell viability was assessed at 48 h after treatment using CCK8 and measured at the optical density of 450 nm, as previously described.^68^

#### Transmission electron microscopy

After specific treatment, cells were collected and centrifuged at 400 g, then resuspended with 0.1 M PBS containing 2.5% glutaraldehyde for 4 h at 4 °C, post-fixed in 1% osmium tetroxide for 2 h at room temperature. Cells were dehydrated using a gradient of ethanol (50–100%) and acetone, embedded in epoxy resin, and polymerized for 48 h at 60 °C. Ultrathin sections (80 nm) were cut and stained with uranyl acetate and lead citrate before transmission electron microscopy (HT7700, HITACHI). Images were obtained using a SlowScan CCD camera and the iTEM software (Ver 01.07, Olympus Soft Imaging Solutions).

#### Flow cytometry analysis for live cells

The numbers of live and dead cells were detected as instructed (ab115347, Abcam). Briefly, cells were collected and washed with Dulbecco’s phosphate buffer saline (DPBS, Thermo Fisher Scientific), then the cells were incubated with 1x live and dead dye for 10 min at room temperature in the dark, and analyzed in the flow cytometer (LSR Fortessa, BD), monitoring the signals from both PE and FITC channels. Data analysis was conducted using FlowJo X software (BD).

#### Glutathione measurements

Cells were collected and prepared for measurement of glutathione using the Glutathione Assay Kit (S0053, Beyotime) according to the manufacturer’s protocol. The GSH and GSSG concentrations were calculated using a standard curve and normalized to the total protein level.

#### Flow cytometry analysis for lipid ROS detection

Cells were collected and washed with DPBS, then incubated with BODIPY 581/591 C11 (D3861, Thermo Fisher Scientific) for 15 min at 37 °C in an incubator. Next, cells were resuspended in 500 μL Hank’s balanced salt solution (Thermo Fisher Scientific), strained through a 70 μm cell strainer (BD), and analyzed in the flow cytometer (LSR Fortessa, BD), monitoring the signals from both on-oxidized C11 (PE channel) and oxidized C11 (FITC channel). Data analysis was conducted using FlowJo X software (BD), calculating the ratios of the mean fluorescence intensity (MFI) of FITC to that of PE.

#### Cellular reactive oxygen species measurements

The cellular ROS levels were measured by H_2_DCFDA (HY-D0940, MedChemExpress). Cells were washed with DPBS and incubated with 5 μM H_2_DCFDA for 30 min at 37 °C in an incubator, and washed three times with DPBS, obtaining images using a fluorescence microscope (Carl Zeiss, Axiovert 200 Basic standard).

#### Flow cytometry analysis for cellular reactive oxygen species measurements

The cellular ROS levels were measured by CellROX Green (C10444, Thermo Fisher Scientific). Cells were collected and washed with DPBS, then the cells were incubated with CellROX Green (5 μM) for 30 min at 37 °C in an incubator. Next, cells were resuspended in 500 μL HBSS, strained through a 70 μm cell strainer (BD), and analyzed in the flow cytometer (LSR Fortessa, BD), monitoring the signals from the FITC channel. Data analysis was conducted using FlowJo X software (BD).

#### Cellular ferrous iron measurements

The cellular ferrous iron levels were measured by FerroOrange (F374, Dojindo). Cells were washed with DPBS and incubated with 1 μM FerroOrange for 30 min at 37 °C in an incubator. The fluorescence intensity (Ex: 543 nm, Em: 580 nm) was determined by a microplate reader (SynergyH1MD, BioTek). The relative fluorescence was normalized by cell number measured by CCK8.

#### Cellular iron detection via inductively coupled plasma mass spectrometry (ICP-MS)

The detection of cellular iron levels via ICP-MS was performed as previously described.^69^ Count the samples to be tested, collect the precipitate, freeze-dry it, then suspend it in 65% nitric acid overnight at room temperature. Treat at 90°C for 20 min, then add an equal volume of 30% hydrogen peroxide and treat at 70°C for 20 min. Determine by ICP-MS (iCAP-6500, Thermo Fisher Scientific) and normalize based on sample counts.

#### Mitochondrial reactive oxygen species measurements

The mitochondrial ROS levels were measured by MitoSOX Red (M36008, Thermo Fisher Scientific). Cells were washed with DPBS and incubated with 500 nM MitoSOX Red for 30 min at 37 °C in an incubator, and resuspended in 500 μL HBSS, strained through a 70 μm cell strainer, and analyzed in the flow cytometer, monitoring the signals from PE Texas Red, analyzing data using FlowJo X software, or obtaining images using a fluorescence microscope, analyzing data using ImageJ software (ver 1.52a, NIH).

#### Mitochondrial membrane potential measurements

The mitochondrial membrane potential levels were measured by MitoProbe JC-1 (M34152, Thermo Fisher Scientific). Cells were collected and incubated with 5 μM JC-1 for 30 min at 37 °C in an incubator, then cells were washed once with DPBS and resuspended in 500 μL HBSS, strained through a 70 μm cell strainer, and analyzed in the flow cytometer, monitoring the signals from both PE and FITC channels. Data was analyzed by FlowJo X software.

#### RNAi and transfection

Cells were seeded into a 6/96 well plate and transfected with siRNA the next day. Following the protocol of Lipofectamine RNAiMAX reagent (13778150, Thermo Fisher Scientific), 10 μM oligonucleotide of siRNA or control siRNA was transfected. After 48 h, the targeted experiments were started. The siRNA was synthesized by GenePharma (Shanghai, China), and the sequences of the siRNA used in the study are listed in the key resources table.

#### Western blot analysis

Cells were collected in cell lysis buffer (P0013, Beyotime) supplemented with the protease inhibitor phenylmethylsulfonyl fluoride (1:100, ST507, Beyotime) and centrifuged at 13,000 × g for 20 min. The supernatant was collected, and the total protein concentration was determined with a BCA protein assay kit (P0011, Beyotime). Equal amounts of protein were separated in 4–20% bis-Tris gels and then transferred to nitrocellulose membranes. Then, the membranes were blocked with 5% skim milk. Next, the membranes were incubated with antibodies overnight at 4 °C. Images were taken by Bio-Rad’s ChemiDoc XRS+ system and then analyzed by ImageJ software (ver 1.52a, NIH). The antibodies used in the study are listed in the key resources table. All original images of the western blots are shown in Figure S6.

#### Bioinformatic analysis

Bioinformatics analysis was performed using web-based bioinformatics tools (http://www.bioinformatics.com.cn/). Venn’s analysis was performed using the data from the Gene Expression Omnibus (GEO) database, including the GSE162550 dataset and the GSE214030 dataset.^35^^,^^36^ Genes with an absolute log_2_ fold change greater than 2 (log_2_FC > 2 or log_2_FC < −2) and a *p*-value less than 0.05 were considered significantly differentially expressed. KEGG and GO pathway enrichment analyses were carried out using R (version 4.4.2) and the clusterProfiler package (version v4.14.6). A set of genes obtained from the Gene Ontology knowledgebase was searched with the keyword oxidative stress without duplicate values. Volcano Plot used data from two datasets to analyze and highlight the DEGs. Using online UALCAN analyzed the expression levels of genes *Mfn1* and *Mfn2* in normal tissues compared to various tumor samples (http://Ualcan.path.uab.edu/analysis).^49^ Online Kaplan-Meier survival analysis was performed to compare OS of Pheochromocytoma and Paraganglioma and Thymoma patients divided by the expression of *Mfn1*, as well as of Sarcoma and Thymoma patients divided by the expression of *Mfn2* in multiple databases (Data related to glioblastoma multiforme were not available in the database) (http://kmplot.com/analysis).^50^

### Quantification and statistical analysis

Data analysis was performed using GraphPad Prism 8.0 software (GraphPad Software). All statistical analyses were conducted based on biological replicates. Data were presented as the means ± SEM. For one-way ANOVA, Tukey’s multiple comparisons test was performed, and for two-way ANOVA, Tukey’s multiple comparisons test was performed. The *p*-value level set for statistical significance is *p* ≤ 0.05.
