# Supplementary material for: HMOX1 drives dihydroartemisinin-sensitized ferroptosis antagonized by mitochondrial fusion
Source: iScience. 2025 Dec 8;29(1):114382. doi: 10.1016/j.isci.2025.114382 (PMC12794436; doi:10.1016/j.isci.2025.114382)
Supplement: Document S1. Figures S1–S6 [file mmc1.pdf]

iScience, Volume *29*

Supplemental information

HMOX1 drives dihydroartemisinin-sensitized ferroptosis antagonized by mitochondrial fusion

Zi-Jie Deng, Jing Zhang, Zhang-Zhong Yang, Qing-Zhang Tuo, and Peng Lei

Supplementary Figures

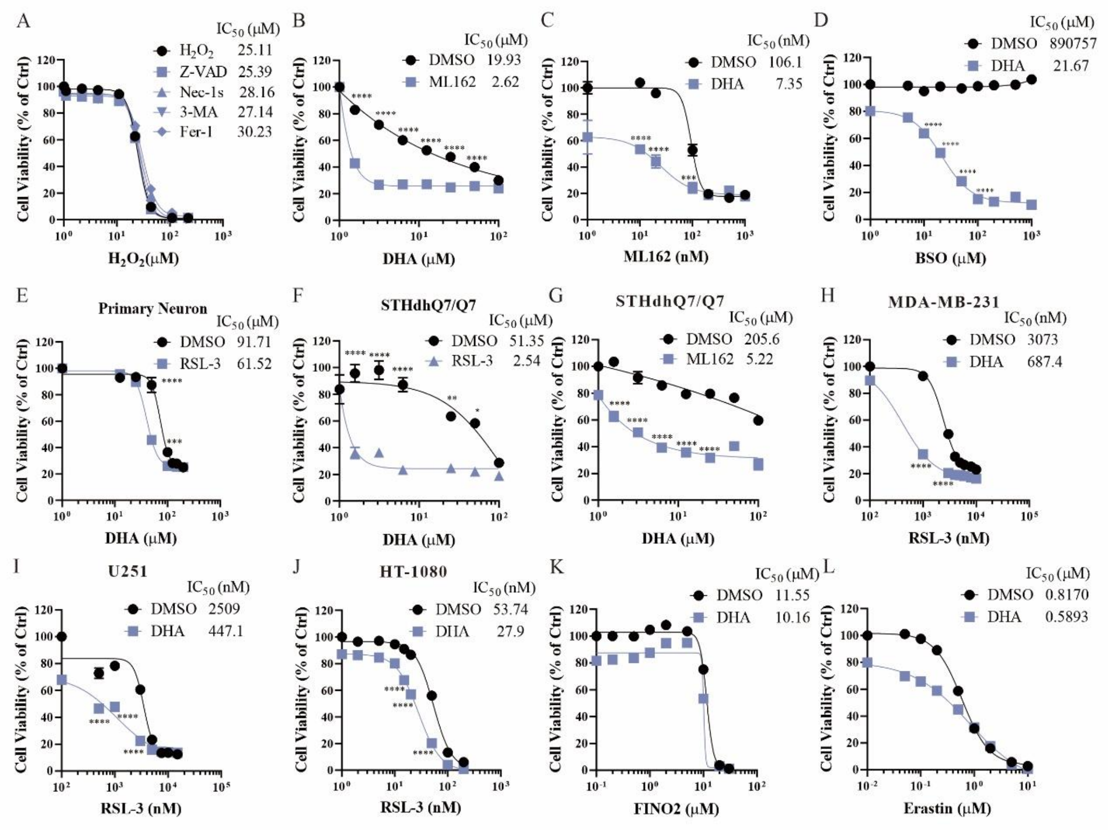

Figure S1 DHA specifically promotes GPX4-dependent ferroptosis related to Figure 1. **(A)** Cell viability of N27 cells treated with different doses of H_2O_2 for 48 hrs in the absence or presence of different cell death inhibitors (20 μM Z-VAD; 10 μM Nec-1s; 1 mM 3-MA; 1 μM Fer-1). **(B)** Cell viability of N27 cells treated with DHA and ML162 (100 nM) for 48 hrs. **(C)** Cell viability of N27 cells treated with ML162 and DHA (1.5 μM) for 48 hrs. **(D)** Cell viability of N27 cells treated with BSO and DHA (1.5 μM) for 48 hrs. **(E)** Cell viability of Primary Neurons treated with DHA and RSL-3 (1 μM) for 48 hrs. **(F)** Cell viability of STHdhQ7/Q7 cells treated with DHA and RSL-3 (100 nM) for 48 hrs. **(G)** Cell viability of STHdhQ7/Q7 cells treated with DHA and ML162 (100 nM) for 48 hrs. **(H)** Cell viability of MDA-MB-231 cells treated with RSL-3 and DHA (1.5 μM) for 48 hrs. **(I)** Cell viability of U251 cells treated with RSL-3 and DHA (1.5 μM) for 48 hrs. **(J)** Cell viability of HT-1080 cells treated with RSL-3 and DHA (1.5 μM) for 48 hrs. **(K)** Cell viability of N27 cells treated with FINO2 and DHA (1.5 μM) for 48 hrs. **(L)** Cell viability of N27 cells treated with Erastin and DHA (1.5 μM) for 48 hrs. Data are means ± SEM, n = 6 wells from one representative of two independent experiments. Two-way ANOVA was performed.

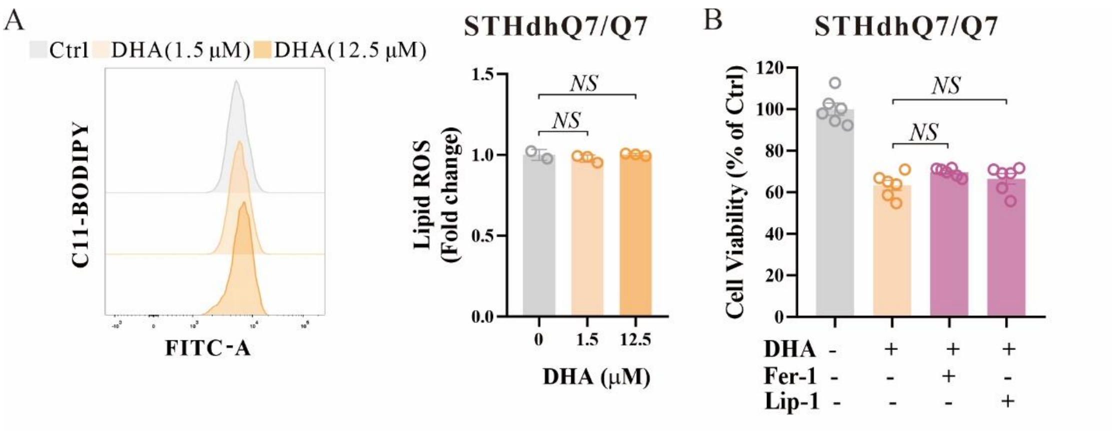

Figure S2 DHA increases sensitivity to ferroptosis by modulating cellular oxidative stress related to Figure 2.

(A) STHdhQ7/Q7 cells were treated with different dose of DHA (0, 1.5 μM, 12.5 μM) for 12 hrs and lipid ROS was detected by C11-BODIPY using flow cytometry. Representative histograms for fluorescence of oxidized C11-BODIPY and the ratio of the mean fluorescence intensity (MFI) of oxidized to reduced C11-BODIPY are shown, n = 3 wells from one representative of two independent experiments. **(B)** Cell viability of STHdhQ7/Q7 cells treated with DHA (12.5 μM) for 48 hrs in the absence or presence of ferroptosis inhibitors (1 μM Fer-1, 1 μM Lip-1). Data are means ± SEM, n = 6 wells from one representative of two independent experiments unless specified. One-way ANOVA was performed.

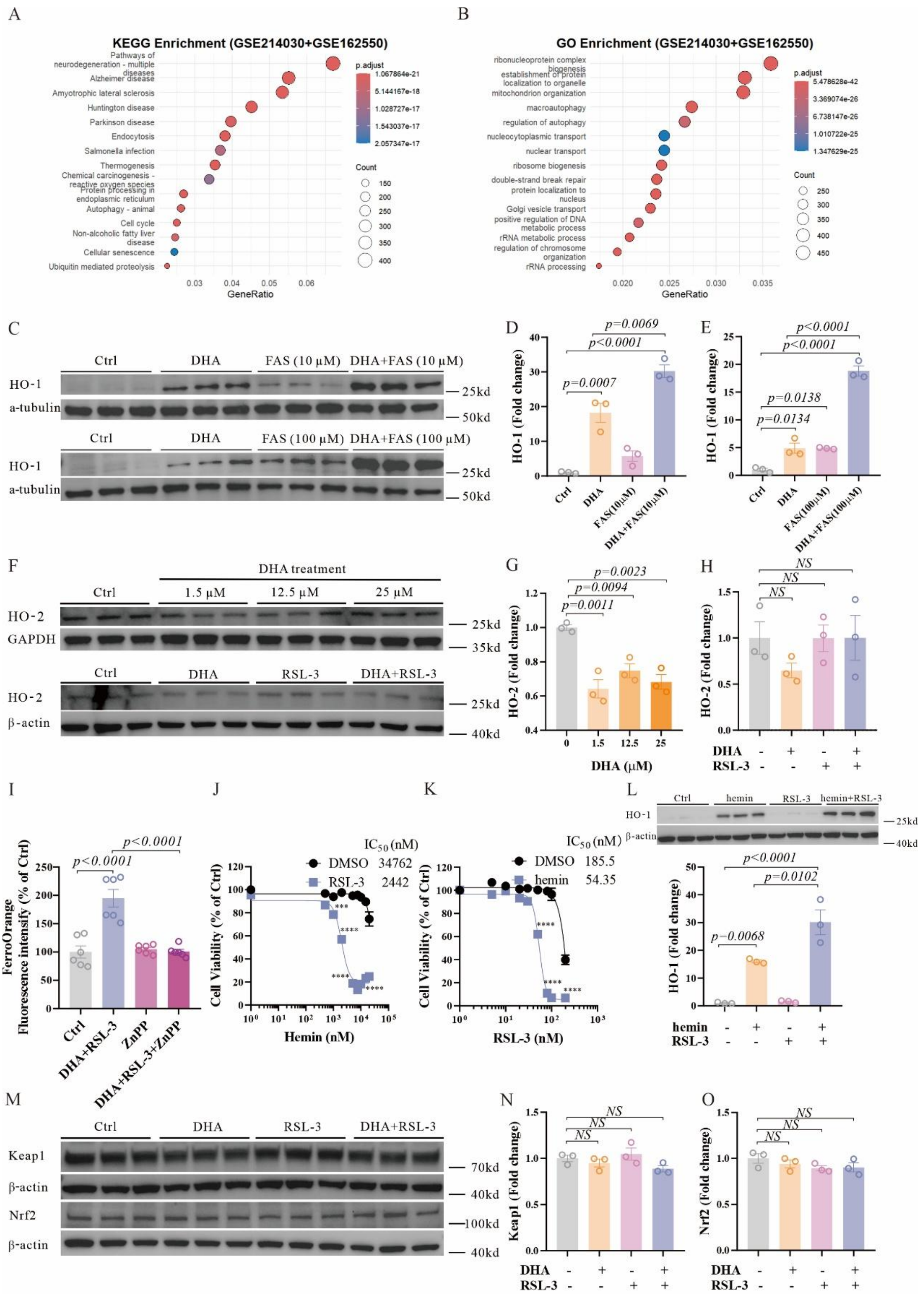

Figure S3 DHA promotes ferroptosis through the activation of HO-1 related to Figure 3.

(A) KEGG pathway enrichment analysis of differentially expressed genes. **(B)** GO enrichment analysis of differentially expressed genes. **(C-E)** Western blot and quantifications of the HO-1 and α-tubulin expression in N27 cells treated with DHA and different doses of FAS (10 μM, 100 μM) for 12 hrs **(F-H)** Western blot and quantifications of the HO-2, GAPDH, and β-actin expression in N27 cells treated with different doses of DHA (0, 1.5 μM, 12.5 μM, 25 μM) for 12

hrs or treated with DHA (1.5 μM) and RSL-3 (100 nM) for 12 hrs. **(I)** The average fluorescence intensity of FerroOrange in N27 cells treated with DHA (1.5 μM) and RSL-3 (100 nM) in the absence or presence of ZnPP (5 μM) for 12 hrs, n = 6 wells from one representative of two independent experiments. **(J)** Cell viability of N27 cells treated with hemin and RSL-3 (100 nM) for 48 hrs, n = 6 wells from one representative of two independent experiments. Two-way ANOVA was performed. **(K)** Cell viability of N27 cells treated with RSL-3 and hemin (6 μM) for 48 hrs, n = 6 wells from one representative of two independent experiments. Two-way ANOVA was performed. **(L)** Western blot and quantifications of the HO-1 and β-actin expression in N27 cells treated with hemin (6 μM) and RSL-3 (100 nM) for 12 hrs. **(M-O)** Western blot and quantifications of the Keap1, Nrf2, and β-actin expression in N27 cells treated with DHA (1.5 μM) and RSL-3 (100 nM) for 12 hrs. Data are means ± SEM, n = 3 wells from one representative of two independent experiments unless specified. One-way ANOVA was performed unless specified.

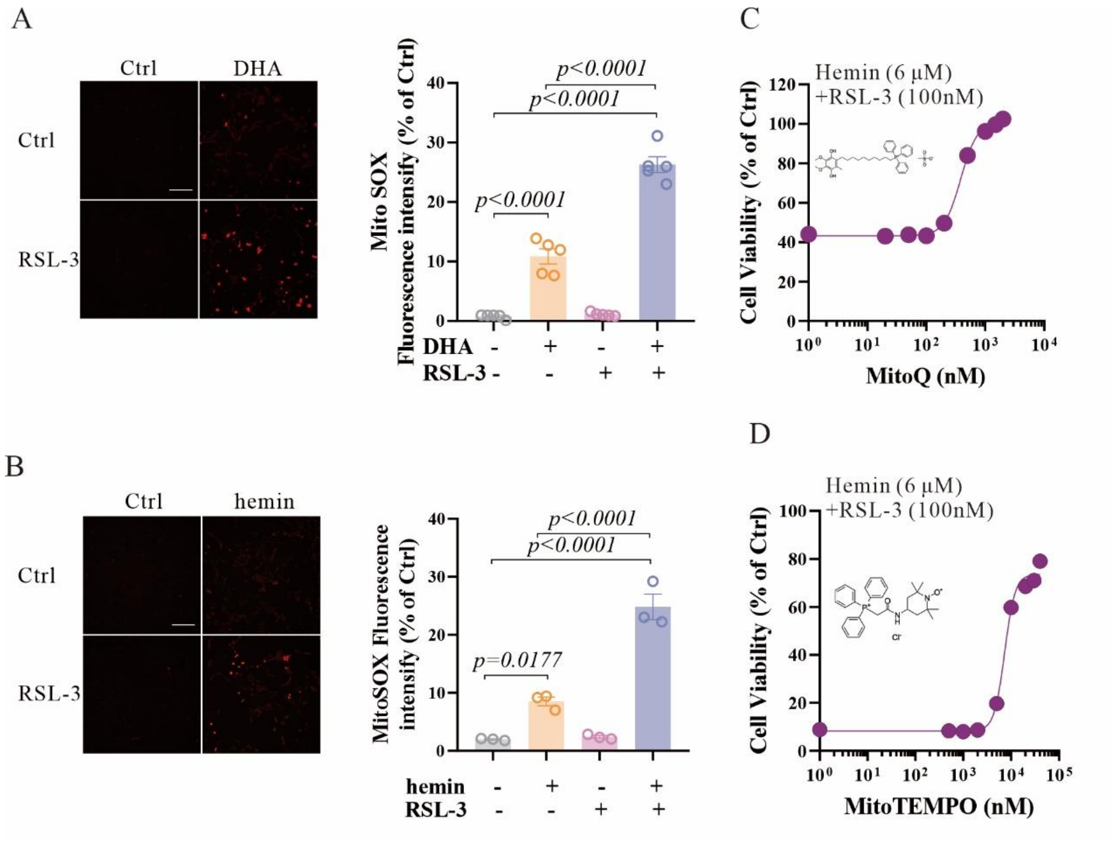

Figure S4 Hemin increases sensitivity to ferroptosis by increasing mitochondrial oxidative stress related to Figure 4.

(A) The representative images of MitoSOX red staining in N27 cells treated with DHA (1.5 μM) and RSL-3 (100 nM) for 12 hrs, and the average fluorescence intensity are shown, n = 5 wells from one representative of two independent experiments. Scale bar, 200 μm, as indicated. **(B)** The representative images of MitoSOX red staining in N27 cells treated with hemin (6 μM) and RSL-3 (100 nM) for 12 hrs, and the average fluorescence intensity are shown, n=3 wells from one representative of two independent experiments. Scale bar, 200 μm, as indicated. **(C, D)** Cell viability of N27 cells after co-treatment of MitoQ or MitoTEMPO with hemin (6 μM) and RSL-3 (100 nM) for 48 hrs, n = 6 wells from one representative of two independent experiments. Data are means ± SEM. One-way ANOVA was performed.

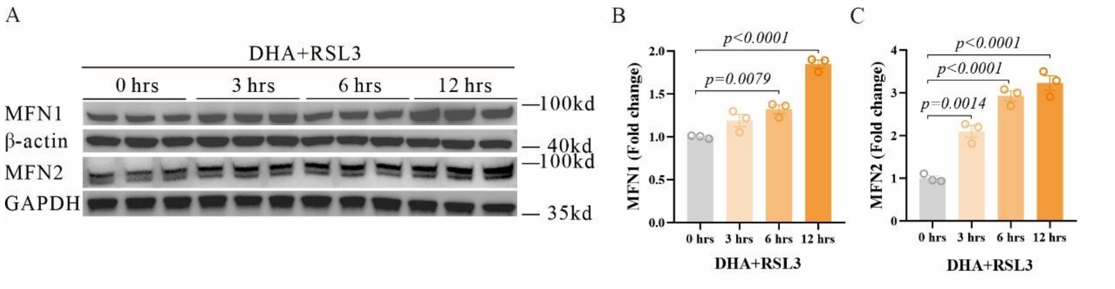

Figure S5 Mitochondrial oxidative stress induced by DHA and RSL-3 activates mitochondrial fusion related to Figure 5.

(A-C) Western blot and quantifications of the MFN1, MFN2, GAPDH and β-actin expression in N27 cells treated with DHA (1.5 μM) and RSL-3 (100 nM) for different timepoint (0 hrs, 3 hrs, 6 hrs and 12 hrs).

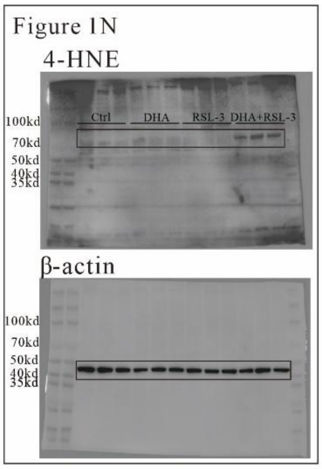
Figure 1N
4-HNE
100kd
70kd
50kd
40kd
35kd
Ctrl
DHA
RSL-3
DHA+RSL-3
β-actin
100kd
70kd
50kd
40kd
35kd

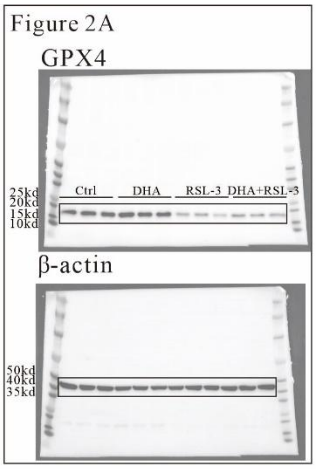
Figure 2A
GPX4
25kd
20kd
15kd
10kd
Ctrl
DHA
RSL-3
DHA+RSL-3
β-actin
50kd
40kd
35kd

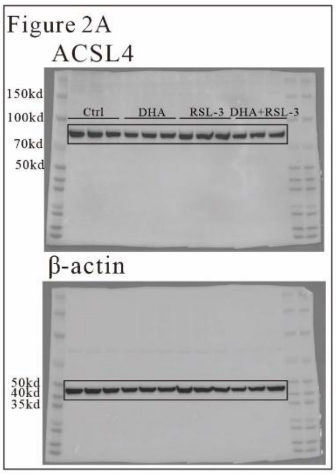
Figure 2A
ACSL4
150kd
100kd
70kd
50kd
Ctrl
DHA
RSL-3
DHA+RSL-3
β-actin
50kd
40kd
35kd

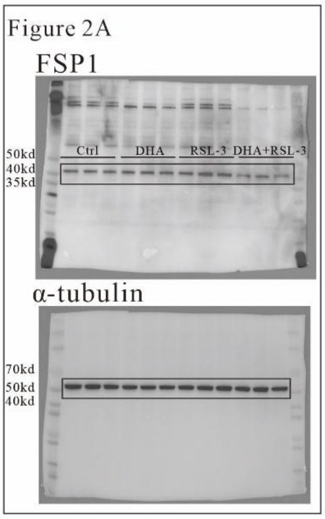
Figure 2A
FSP1
50kd
40kd
35kd
Ctrl
DHA
RSL-3
DHA+RSL-3
α-tubulin
70kd
50kd
40kd

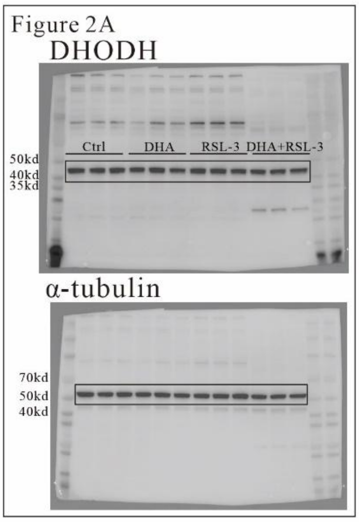
Figure 2A
DHODH
50kd
40kd
35kd
Ctrl
DHA
RSL-3
DHA+RSL-3
α-tubulin
70kd
50kd
40kd

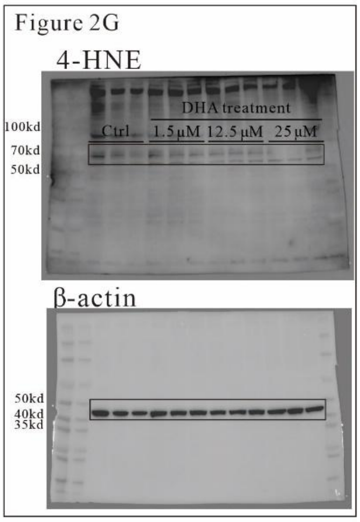
Figure 2G
4-HNE
DHA treatment
100kd
70kd
50kd
Ctrl
1.5μM
12.5μM
25μM
β-actin
50kd
40kd
35kd

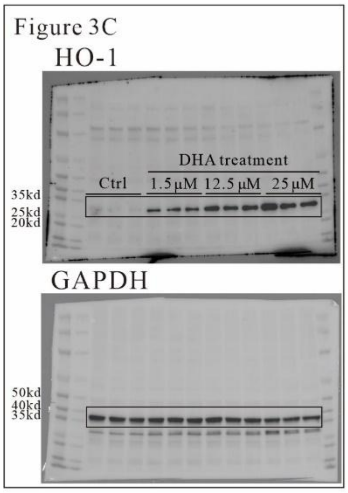
Figure 3C
HO-1
DHA treatment
35kd
25kd
20kd
Ctrl
1.5μM
12.5μM
25μM
GAPDH
50kd
40kd
35kd

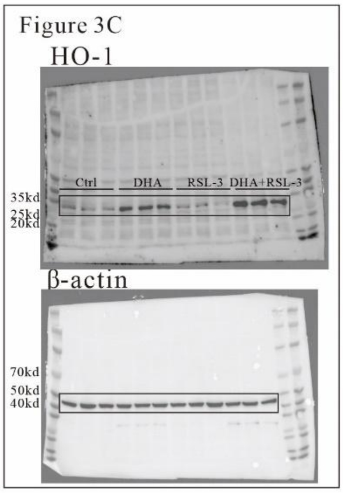
Figure 3C
HO-1
35kd
25kd
20kd
Ctrl
DHA
RSL-3
DHA+RSL-3
β-actin
70kd
50kd
40kd

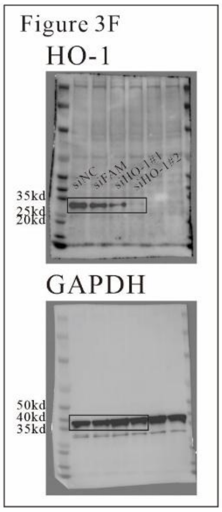
Figure 3F
HO-1
siNC
siFAM
siHO-1#1
siHO-1#2
35kd
25kd
20kd
GAPDH
50kd
40kd
35kd

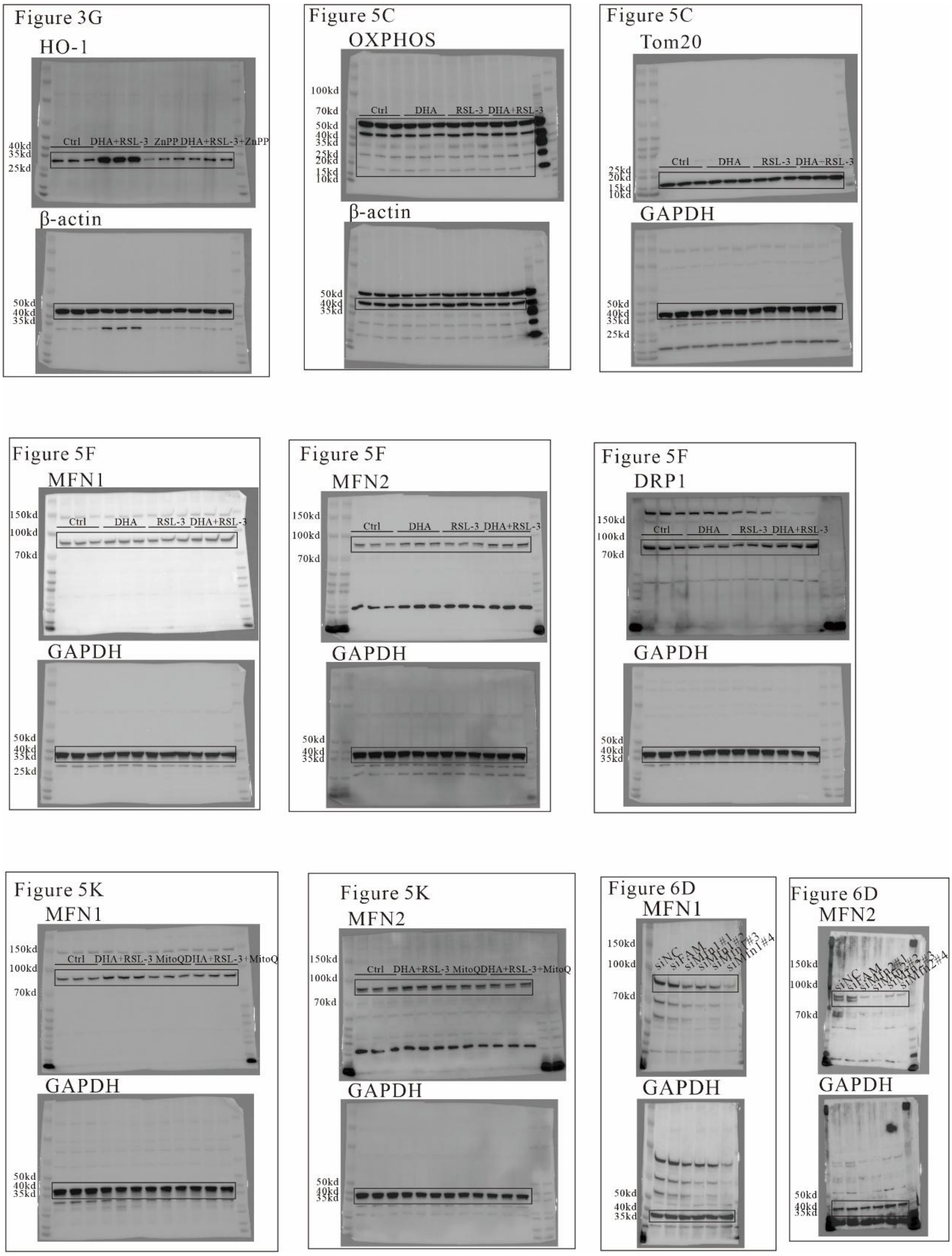
Figure 3G
HO-1
Ctrl
DHA+RSL-3
ZnPP
DHA+RSL-3+ZnPP
40kd
35kd
25kd
β-actin
50kd
40kd
35kd
Figure 5C
OXPHOS
100kd
70kd
50kd
40kd
35kd
25kd
20kd
15kd
10kd
Ctrl
DHA
RSL-3
DHA+RSL-3
β-actin
50kd
40kd
35kd
Figure 5C
Tom20
25kd
20kd
15kd
10kd
Ctrl
DHA
RSL-3
DHA+RSL-3
GAPDH
50kd
40kd
35kd
25kd
Figure 5F
MFN1
150kd
100kd
70kd
Ctrl
DHA
RSL-3
DHA+RSL-3
GAPDH
50kd
40kd
35kd
25kd
Figure 5F
MFN2
150kd
100kd
70kd
Ctrl
DHA
RSL-3
DHA+RSL-3
GAPDH
50kd
40kd
35kd
Figure 5F
DRP1
150kd
100kd
70kd
Ctrl
DHA
RSL-3
DHA+RSL-3
GAPDH
50kd
40kd
35kd
Figure 5K
MFN1
150kd
100kd
70kd
Ctrl
DHA+RSL-3
MitoQ
DHA+RSL-3+MitoQ
GAPDH
50kd
40kd
35kd
Figure 5K
MFN2
150kd
100kd
70kd
Ctrl
DHA+RSL-3
MitoQ
DHA+RSL-3+MitoQ
GAPDH
50kd
40kd
35kd
Figure 6D
MFN1
150kd
100kd
70kd
GAPDH
50kd
40kd
35kd
Figure 6D
MFN2
150kd
100kd
70kd
GAPDH
50kd
40kd
35kd

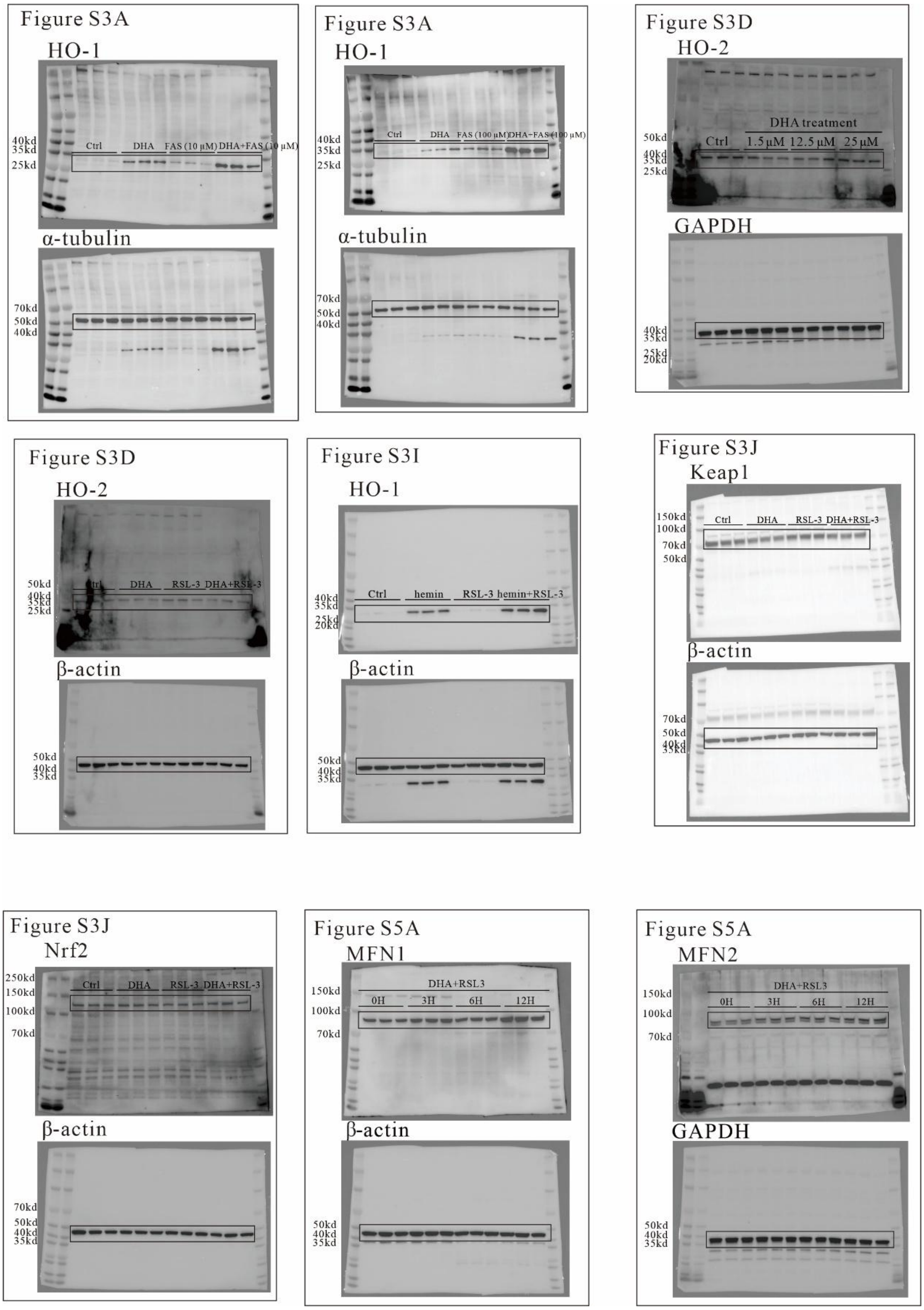

Figure S6 Scans of uncropped blots.
